# Development of a Novel 2-DOF Rotary–Linear Piezoelectric Actuator Operating under Hybrid Bending–Radial Vibration Mode

**DOI:** 10.3390/mi12060728

**Published:** 2021-06-21

**Authors:** Andrius Čeponis, Dalius Mažeika, Daiva Makutėnienė

**Affiliations:** 1 Department of Engineering Graphics, Faculty of Fundamental Sciences, Vilnius Gediminas Technical University, Sauletėkio al. 11, LT-10223 Vilnius, Lithuania; daiva.makuteniene@vilniustech.lt; 2 Department of Information Systems, Faculty of Fundamental Sciences, Vilnius Gediminas Technical University, Sauletėkio al. 11, LT-10223 Vilnius, Lithuania; dalius.mazeika@vilniustech.lt

**Keywords:** piezoelectric actuator, 2-DOF rotation, 2-DOF linear motion

## Abstract

The paper presents a numerical and experimental investigation of a novel two degrees of freedom (2-DOF) piezoelectric actuator that can generate rotary motion of the sphere-shaped rotor as well as induce planar motion of the flat stage. The actuator has a small size and simple design and can be integrated into a printed circuit board (PCB). The application field of the actuator is small-dimensional and high-precision positioning systems. The piezoelectric actuator comprises three rectangular bimorph plates joined with arcs and arranged by an angle of 120 degrees. A high-stiffness rod is glued on the top surface of each bimorph plate and is used to rotate the rotor or move flat stage employing contact friction force. Three U-shaped structures are used for the actuator clamping. 2-DOF rotational or planar movement is obtained by applying a harmonic or asymmetric electrical signal. The operation principle of the actuator is based on the superposition of the B_20_ out-of-plane bending mode of the bimorph plates and the B_03_ radial vibration mode of the ring. Design optimization has been performed to maximize amplitudes of contact point vibration. A prototype of the actuator was made, and a maximum rotation speed of 795.15 RPM was achieved while preload of 546.03 mN was applied. The linear velocity of 36.45 mm/s was obtained at the same preload force. Resolution measurement showed that the actuator can achieve an angular resolution of 17.48 µrad and a linear resolution of 2.75 µm.

## 1. Introduction

Piezoelectric actuators and motors are widely used in high-precision systems such as laser beam position and focusing systems, scanning and manipulation systems, robotics, etc. [1,2,3]. Wide application of piezoelectric actuators and motors is affected by the following advantages as nanoscale or microscale resolution, self-locking, high volume-to-torque ratio, gear free-motion transfer, low backlash or no backlash when motion direction is changing, magnetic-field-free operation, etc. [4,5]. Usually, piezoelectric actuators and motors provide a single degree of freedom linear or rotational motion [6,7]. Therefore, multidegree of freedom systems comprise several actuators or motors. As a result, the mechanical systems have a complex structure, occupy larger space, and control becomes more complicated. A separate driver and controller is required for each motor because the resonant frequencies of the transducers are different. In addition, it is difficult to achieve high resolution and adapt such systems for specific applications [8,9]. The piezoelectric actuators and motors generating several degrees of freedom have smaller overall sizes, are simple to manufacture, and provide the possibility of obtaining different types of motion [10]. Multi-DOF actuators allow one to simplify the overall structural design of the mechanical system and reduce the size and space allocation of the system. In addition, it allows one to extend the scope of the actuator application [11,12].

The structural design of multi-DOF actuators in most cases is based on simple structures such as piezoelectric rings, cylinders, bimorph plates, or discs that compose one mechanical system and are driven by several electric signals [13]. Usually, one electrical signal per degree of freedom is needed to drive a multi-DOF actuator; therefore, the driving circuit becomes more sophisticated than a single-DOF actuator [14,15]. Dynamic characteristics of the multi-DOF actuators depend on the design and operating principle. Resonant-type ultrasonic actuators are excited by harmonic signal and achieve higher velocity and torque, while inertial type actuators are characterized by higher resolution [16,17]. There are several multi-DOF actuators that can achieve high velocity and few micron range resolution. Most of the multi-DOF actuators can provide one type of motion, i.e., rotational or linear [18,19,20]. However, considering the demand of modern positioning and manipulation systems, dual-function actuators are developed. A piezoelectric dual-function rotary–linear multi-DOF actuator was developed for high-resolution microscopy applications [20]. The actuator was composed of rotary and linear actuators placed on each other. The actuators operates in nonresonant mode and achieves high resolution. However, the actuator has a complicated design and limited scalability options. A more advanced dual-function rotary–linear 2-DOF piezoelectric actuator was proposed by Mashimo and Touama [21]. The design of the actuator is based on a small-size hollowed cube and cylindrical shaft. The actuator operates at two different deformation modes and can move the shaft linearly and rotate it about the longitudinal axis. However, in order to drive the actuator, four excitation signals are needed, and the clamping of the actuator is complicated. A dual-function 2-DOF actuator was developed that provides linear-rotary motion of the slider placed on a cylindrical shaft [22,23]. However, the linear motion of the slider is limited by the length of the shaft, and the clamping of the actuator can be challenging. There are screw-type piezoelectric actuators that can provide coupled or independent rotary–linear motion of the shaft [24]. This type of actuator has a simple and scalable design, but some of these type actuators have a restriction for one direction of the motion by the structural design, thus reducing the number of degrees of freedom.

In this paper, a novel design of a 2-DOF rotary–linear actuator was proposed that can achieve high velocity and micrometric resolution. The flat design of the actuator allows one to integrate it into PCB. A single harmonic signal is needed to drive the actuator. Control of motion direction is implemented by a simple digital switch box.

The rest of the paper is organized as follows: Section 2 describes the structure, dimensions, and operation principle of the actuator. In addition, excitation schematics of the electrodes are described. Section 3 presents the results of numerical modelling and design optimization. Section 4 provides measured characteristics of the actuator, i.e., rotational and linear speed for different applied voltages and measurement results of angular and linear resolution. Finally, Section 5 concludes this work.

## 2. Design and Operation Principle of the Actuator

**Figure 1:**
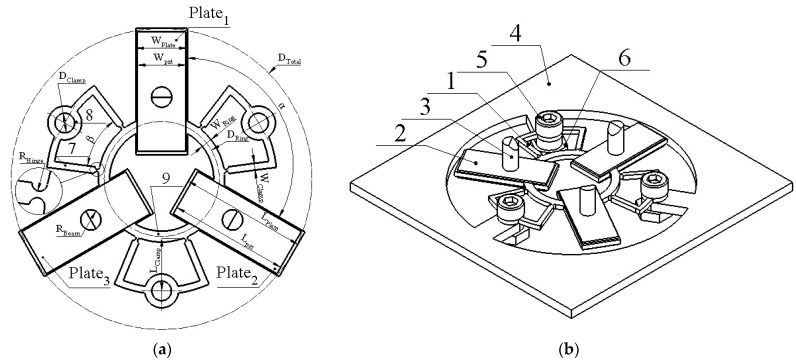
Design of the actuator; (**a**)—sketch of the stator; (**b**)—view of the actuator clamped into the PCB; 1—stator; 2—piezo ceramic plates; 3—cylinder rod; 4—actuator clamping frame (PCB); 5—fixing bolts; 6—fixing nuts; 7—U-shaped supports; 8—clamping spot; and 9—connecting arc.

The piezoelectric actuator is composed of three stainless-steel plates joined with the arcs and arranged by an angle of 120 degrees (Figure 1a). Two piezoceramic plates are glued on the top and bottom surfaces of the steel plate and form piezoelectric bimorphs. Polarization of the piezoelectric plates is aligned with thickness and has the same direction in the assembled bimorph the out-of-plane bending mode of the bimorph plates is employed for motor operation; therefore, the junction point of the arcs and bimorph plates coincide with the end of the nodal line of the bimorph vibrations. Three U-shaped supports are used to clamp the actuator. Supports are connected to the arcs’ outer surface via flexible hinges to reduce structural damping of the stator vibrations (Figure 1b). The junction points of the supports coincide with the nodes of the radial vibrations of the arcs in order to reduce structural damping of the stator vibration. The lengths of the supporting beams are chosen so that the resonant frequency of the first bending mode of the beams coincides with the operating frequency of the motor.

The actuator is clamped into the PCB housing frame using three bolts. A high-stiffness cylindrical rod is glued on the top surface of each bimorph plate. The spherical rotor or flat sliding stage is placed on the rods, and the rotational or linear motion of the rotor or slider is achieved. The chamfers are designed at the free end of the high-stiffness rods to increase the contact area between rods and spherical rotor. The sketch of the stator is presented in Figure 1a, while geometrical parameters are listed in Table 1.

The operation of the actuator is based on the superposition of the B_20_ bending vibration mode of the bimorph plate and the B_03_ radial vibration mode of the ring. Describing the operation of the motor, we assumed that arcs joined with the bimorph plates form a ring-type structure. The bimorph plate bends in the out-of-plane direction when the B_20_ bending mode is excited and generates minor in-plane motion as well. Therefore, the radial vibrations of the ring are excited when the radial and bending vibration modes have the same resonant frequencies. Such stator vibrations create curvilinear vibrations of the contact point in the plane perpendicular to the stator plane. In order to increase amplitudes of the contact point vibrations, the dimensions of the bimorph plates and the ring must be optimized so that resonant frequencies of the B_20_ bending mode of the bimorph plates and the B_03_ radial vibration mode of the ring coincide. In addition, the position of the rods on the top surface of the plate is moved by distance ξ from the antinode of the bending standing wave. It allows for the increase in contact point vibration amplitudes in the radial direction. A sine wave electrical signal is applied to the piezoelectric bimorph electrodes while the metal layer is grounded This type of circuit is used to excite out-of-plane bending vibrations of the bimorph and radial oscillation of the ring.

**Figure 2:**
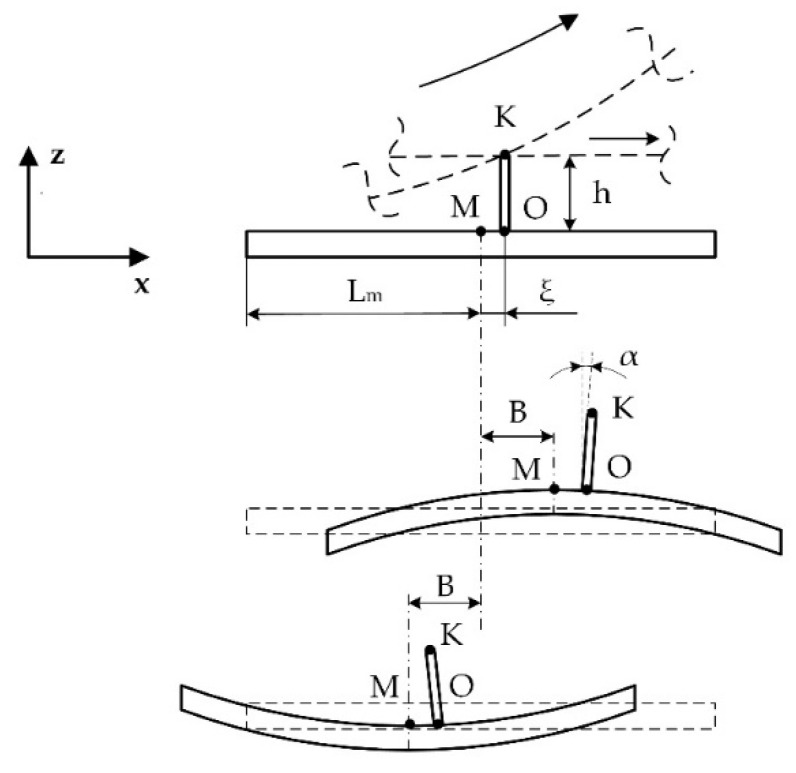
Positions of the bimorph plates during actuator operation.

A simplified model of the bimorph vibrations is shown in Figure 2, where motion decomposition is presented. It was assumed that bending vibrations have a sine wave modal shape. Contacting point *K* is located on the top of the cylindrical rod and is placed at point *O*, which is moved by distance *ξ* from antinode *L_m_*. The position of the contact point *K* along the *x* axis can be written as:

(1) $$x_{K}=L_{m}+\xi$$

When bending vibrations of the plate are excited, then modal displacement of the plate in the direction of *z* axis can be expressed as:

(2) $$z(x)=A\sin\left(\frac{2\pi }{\lambda }x\right)$$

where *A* is the vibration amplitude of bending vibrations of the bimorph plate and *λ* is the wavelength. Vibration amplitude *A* is a function of excitation voltage, piezoelectric constant, width, and the thickness of the metal plate and piezo ceramic layer, as well as properties of the materials [2]. When the plate deforms, the rod is rotated by an angle of α, and the displacement of the contact point *K* in the direction of the *x* axis is induced. Radial vibrations of the ring generate contact point displacement along the *x* axis as well; therefore, the point *K* total displacement in the direction of *x* axis can be written as:

(3) $$\Delta x_{K}=h\sin\left(\alpha \right)+B$$

where *B* is the vibration amplitude of radial vibrations and *h* is the height of the rod. Vibration amplitude *B* is a function of external and internal radius, material properties, and excitation force of the ring. Based on geometric relations (Figure 1b), the displacement of point *K* in the direction of *z* axis is:

(4) $$\Delta z_{K}=A\sin\left(\frac{2\pi }{\lambda }x_{K}\right)-h\left(1-\cos\left(\alpha \right)\right)$$

Angle *α* can be expressed as follows:

(5) $$\alpha \approx \frac{\partial z_{K}}{\partial x}=A\frac{2\pi }{\lambda }\cos\left(\frac{2\pi }{\lambda }x_{K}\right)$$

The vibration amplitude *A* is very small compared to the wavelength; therefore, angle *α* is very small too. Based on that, it can be assumed that sin(*α) ≈ α* and cos(*α) ≈* 1; therefore, the displacement of the point *K* can be written as follows:

(6) $$\{\begin{array}{l} \Delta x_{K}=hA\frac{2\pi }{\lambda }\cos\left(\frac{2\pi }{\lambda }x_{K}\right)+B\\ \Delta z_{K}=A\sin\left(\frac{2\pi }{\lambda }x_{K}\right) \end{array}$$

Assuming that the bimorph plate operates at the bending mode when *λ = 2L*, then the total amplitude of the point *K* vibrations can be expressed as:

(7) $$U_{K}=\sqrt{A^{2}\left(\left(\frac{h^{2}\pi^{2}}{L^{2}}-1\right)\cos^{2}\left(\pi \left(1+\frac{\xi }{L}\right)\right)+1\right)+2AB\frac{h\pi }{L}\cos\left(\pi \left(1+\frac{\xi }{L}\right)\right)+B^{2}}$$

It can be seen that contact point displacements in *x* direction depend on the height of the rod and amplitude of bending and radial vibrations. In addition, rod position on the top surface of the bimorph is important, because as distance ξ from the antinode increases, the displacement in the *x* direction increases but decreases in the z direction. Therefore, the rod position must be optimized in order to maximize total displacement amplitudes of contact point *K*. 

**Figure 3:**
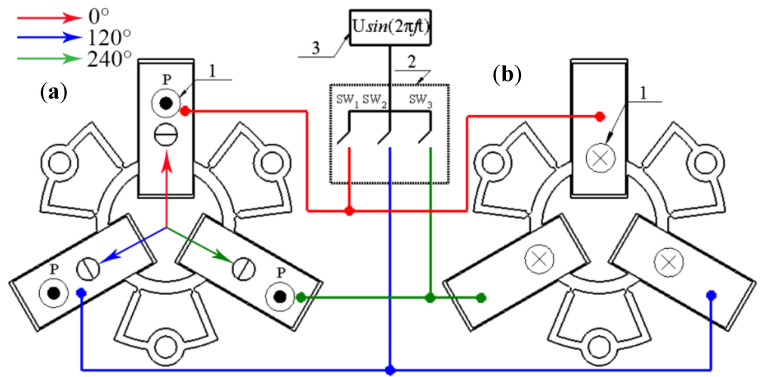
Excitation schematics of the actuator; (**a**)—top view; (**b**)—bottom view; 1—direction of piezo ceramic plate polarization; 2—switch box; and 3—excitation signal source.

The design and operation principle of the actuator provides the possibility to obtain planar or rotation motion of the stage or rotors employing the same vibration mode of the actuator that composes bending mode of the plate (B_20_) and radial mode of the ring (B_30_). The proposed actuator allows one to achieve controllable rotation of the rotor or planar motion of the slider when the harmonic electric signal is applied to the corresponding piezoelectric plates. The excitation schematics and direction of the induced linear or rotary motion are given in Figure 3 and Table 2.

It can be seen that control of the motion direction can be implemented using digitally controlled switching of a single harmonic signal. Special trajectory-planning algorithms can be used to generate the required planar or rotational motion of the output link [25]. The algorithms include variation in amplitudes of electric signals, duration time, and sequences of switching control. High displacement and rotation resolution of the motor can be achieved applying burst type electric signal. In order to increase the output velocity, force, and torque of the actuator, switching between pairs of the bimorph plates can be implemented.

## 3. Numerical Study of the Actuator

Numerical modeling of the actuator was performed to optimize dimensions of the actuator, find the optimal location of the rod, and make frequency response analysis. The finite element model of the actuator was built using Comsol 5.4. The following materials were used in the model: i.e., stainless steel was used for the passive layer of the stator, PIC181 (PI Ceramic GmbH, Germany) material properties were used for piezo ceramic plates, and alumina oxide was used for cylindrical rods. Material properties are listed in Table 3. Boundary conditions of the model were set as follows: clamping spots were fixed rigidly, while electrical boundary conditions were set as it is shown in Figure 3.

Optimization of actuator dimensions was carried out with the goal to match resonant frequencies of the B_20_ bending mode of the bimorph plate and the B_03_ radial mode of the ring by parametric. The diameter of the ring and the length of metal plates were selected as design variables. The optimization problem is described below:

(8) $$\underset{L,D}{\min}(\left|f_{B20}(L_{Plate},D_{Ring})-f_{B03}(L_{Plate},D_{Ring})\right|);$$

subject to:

(9) $$L_{Plate}^{\min}\leq L_{Plate}\leq L_{Plate}^{\max}$$

(10) $$D_{Ring}^{\min}\leq D_{Ring}\leq D_{Ring}^{\max}$$

(11) $$f_{\min}\leq f\leq f_{\max}.$$

where *L_Plate_* is the length of metal plates; $D_{Ring}^{}$ is the diameter of ring; *f_B20_* is the resonant frequency of B_20_ out of the plane bending mode of the bimorph plate; *f_B03_* is the resonant frequency of B_03_ radial mode of the ring; $L_{Plate}^{\min}$ and $L_{Plate}^{\max}$ are the minimum and the maximum lengths of metal plates, respectively; $D_{Ring}^{\min}$ and $D_{Ring}^{\max}$ are the minimum and the maximum diameters of ring; *f* is the resonant frequency of the actuator; *f_min_* and *f_max_* are minimum and maximum limits of the analyzed frequency range. $L_{Plate}^{\min}$ and $L_{Plate}^{\max}$ were set to 8.4 and 10.5 mm, respectively. The minimum and maximum values of ring center diameter were as follows: $D_{Ring}^{\min}$ = 7.3 mm and $D_{Ring}^{\max}$ = 9.2 mm. The linear search was used to find the optimal value of the objective function. The step size of ring diameter and plate length variation was 0.1 mm for course analysis, while the step of 0.01 mm was used to make fine resolution analysis in the narrower range in order to get a more precise value. The frequency range from *f_min_ =* 35 kHz to *f_max_* = 52 kHz and step size of 2.5 Hz were determined. Frequency response analysis was performed applying a voltage of 100 V_p-p_ for the actuator excitation.

**Figure 4:**
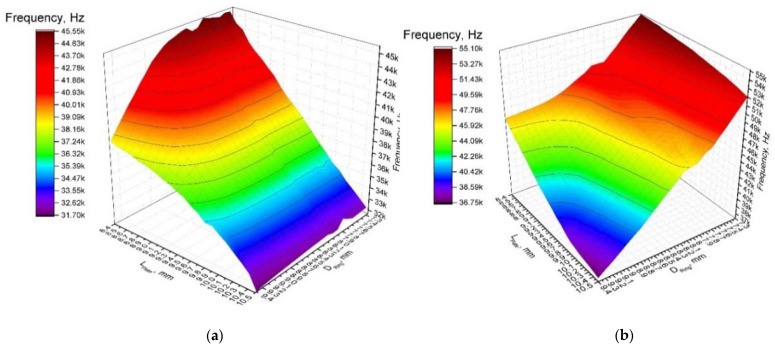
Dependence of resonant frequency from the length of the plate and diameter of the ring: (**a**)—bending mode (B_20_) and (**b**)—radial mode (B_03_).

**Figure 5:**
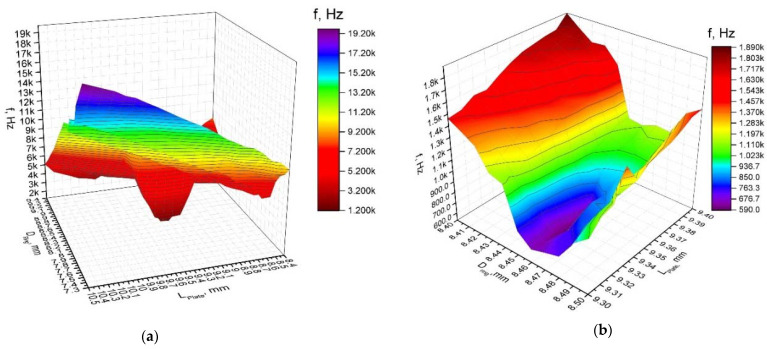
Modulus of the difference between resonant frequencies versus length of the plate and diameter of the ring when step size of 0.1 mm (**a**), and 0.01 mm (**b**) is used.

First of all, dependencies of the resonant frequencies of the bending vibration mode B_20_ and radial vibration mode B_03_ from the length of the plate and diameter of the ring were calculated (Figure 4a,b). It must be pointed out that these calculations cannot be fully automated, because the sequence of the vibration modes changes in the determined frequency range when dimensions of the plate and ring vary within specified ranges. Analysis of the obtained results revealed that the resonant frequency of the bimorph plate strongly depends on the plate length, and frequency decreases when the length of the plate increases. On the other hand, the resonant frequency of the radial vibration mode B_03_ increases when the length of the plate and diameter of the ring decreases. The modulus of difference between resonant frequencies of aforementioned vibration modes, i.e., objective function, was calculated at the next step of the optimization study. Results are shown in Figure 5a, when a step size of 0.1 mm was applied. Analyzing the results, it can be seen that the objective function reaches minimum value when plate length and ring diameter are within the range of 9.30–9.40 mm and 8.40–8.50 mm, respectively. Therefore, a study with a step size of 0.01 mm was performed in order to get more precise results (Figure 5b). This split analysis when coarse and fine steps were used allowed us to save the computational time.

Analyzing obtained result, it can be seen that the objective function has a minimum value of 520 Hz at the following dimensions: *L_Plate_* = 9.33 mm and $D_{Ring}^{}$ = 8.46 mm. The resonant frequency of the actuator is 42.91 and 42.39 kHz of the B_20_ bending and B_03_ radial modes, respectively.

**Figure 6:**
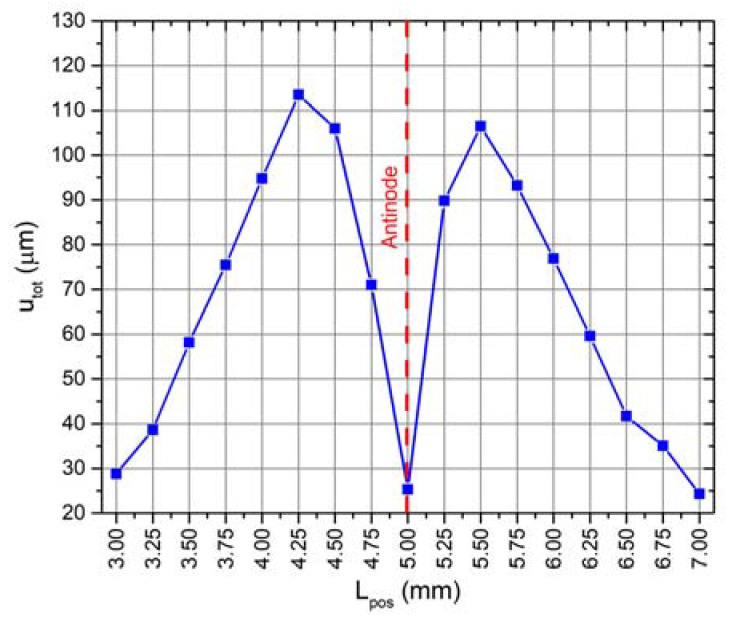
Dependence of the contact point total displacement amplitude from the position of the rod.

Optimization of the cylindrical rod position along the symmetry axis of the bimorph plate was performed with the goal to increase amplitudes of contact point vibrations. The analyzed actuator has a symmetrical structure; therefore, only one bimorph plate with the rod was analyzed. The total vibration amplitude of the contact point was defined as an objective function. The optimization problem is written as:

(12) $$\underset{L_{pos}}{\max}\left(\sqrt{u_{x}^{2}\left(L_{pos}\right)+u_{z}^{2}\left(L_{pos}\right)\;}\right)$$

subject to:

(13) $$L_{pos}^{\min}\leq L_{pos}\leq L_{pos}^{\max}$$

(14) $$f_{B20}^{}=f_{B03}^{}$$

where *u_x_* and *u_z_* are the displacement amplitudes in the direction of *X* and *Z* axis, respectively; *L_pos_* is the position of the cylindrical rod along the axis of symmetry of the bimorph plate measured from the inner edge of the plate; $L_{pos}^{\min}$ and $L_{pos}^{\max}$ are the lower and upper limits, respectively. The following limit values were used: $L_{pos}^{\min}$ = 3 mm and $L_{pos}^{\max}$ = 7 mm, while the increment step was 0.25 mm. The constraint defined in Equation (14) ensures that the mode superposition condition is fulfilled. The dependence of the objective function from rod position is shown in Figure 6.

It can be seen that graph of total displacement amplitudes has two peaks, i.e., 113.5 and 106.1 µm, obtained when the position of the cylindrical rod is equal to 4.25 and 5.5 mm. The peaks have different values, because the bimorph plate has an asymmetrical modal shape due to the asymmetrical clamping, i.e., the bimorph plate has a junction with the ring at just one side (Figure 1). The total displacement curve has a minimum point at the position of 5.0 mm. The displacement amplitude of the contact point is equal to 25.3 µm at this position (Figure 6). It corresponds to the position of the antinode of the B_20_ bending mode of the bimorph plate. Analysis of the displacement amplitude projections when the rod is placed in antinode showed that the contact point vibrates mainly in *Z* axis direction, while the displacement in the X and Y directions is 1.8% and 0.75% of the total displacement, respectively. L_pos_ of 4.25 mm was chosen as the optimal position of the rod due to the highest displacement amplitude. The optimization of the rod position allowed one to increase the contact point displacement amplitude up to 22.7% compared to the previous study results. A summary of the results is given in Table 4.

**Figure 7:**
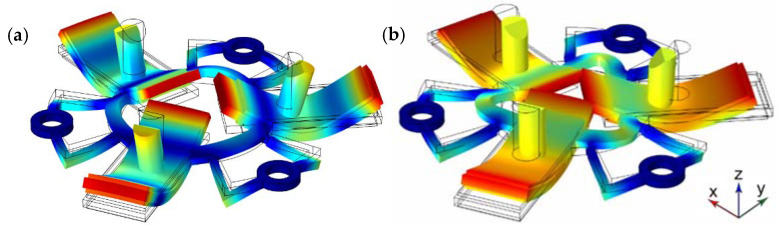
The modal shape of the stator at the frequency of 42.80 (**a**) and 43.39 kHz (**b**).

The next step of the numerical investigation was to perform modal analysis of the actuator. The optimization of the actuator dimensions allowed one to achieve a difference of the resonant frequencies equal to 590 Hz. The resonance frequency of the B_20_ bending mode of the bimorph plates and B_03_ radial mode of the ring is 42.80 and 43.39 kHz, respectively. The modal shapes are shown in Figure 7. It can be seen that modal shapes are similar, and the bending mode of the bimorphs induces radial vibrations of the ring, and the B_03_ radial vibration mode composes B_20_ bending mode of the bimorph plates. In addition, it must be noted that the junction points of the U-shaped clamping structure and the ring are located precisely at nodal points of B_03_ vibration mode and vibrate in the first bending mode. Harmonization of the vibration modes of the individual structural components of the actuator makes it possible to achieve low structural damping and increase amplitudes of vibrations.

**Figure 8:**
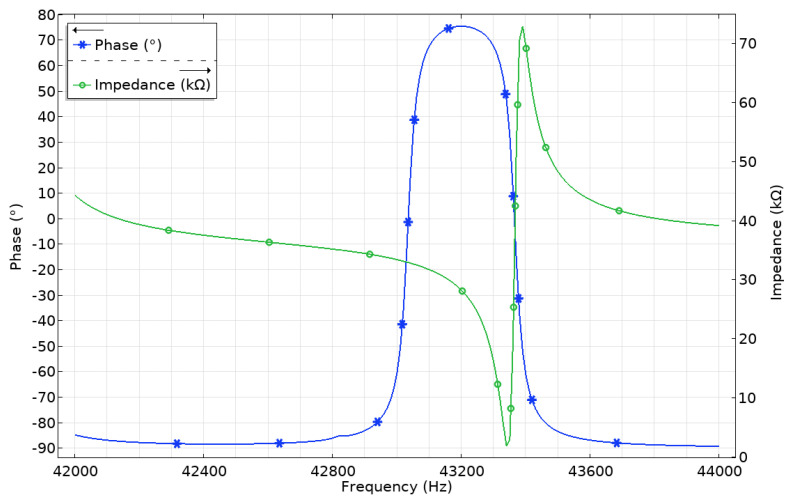
Impedance-frequency and phase-frequency characteristics of the actuator.

The further step of the numerical study was to analyze impedance and phase characteristics of the actuator in the frequency range from 42 to 44 kHz with the step of 5 Hz (Figure 8). It can be seen that the lowest impedance value of 1.34 kΩ is obtained at the resonant frequency of 43.345 kHz. Moreover, the calculated mechanical quality factor of the actuator is *Q_m_* = 2422.7, while the effective coupling coefficient is *k_eff_* = 0.047. This means that the actuator has low mechanical loss at the operating frequency of the actuator.

**Figure 9:**
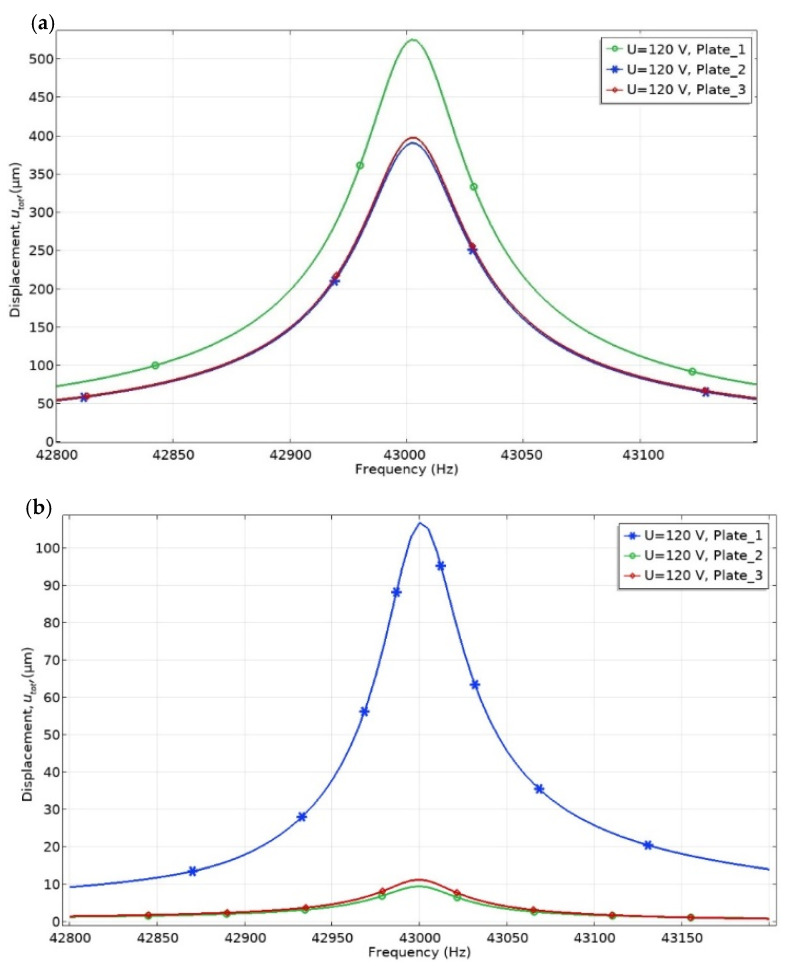
Displacement amplitude versus frequency of the three contact points when one bimorph plate is excited with no load (**a**) and the mechanical load is applied (**b**).

Numerical analysis of the mechanical characteristics of the actuator was performed as the next step of the numerical study. Vibrations of the three contact points were analyzed when only one bimorph plate was excited. This excitation regime is the most commonly used during actuator operation. The frequency range was set from 42.8 to 43.4 kHz, with a step resolution of 2.5 Hz. Figure 9 shows the displacement amplitude change in the frequency domain, when the excitation voltage of 120 V_p-p_ is applied on the single bimorph plate (Plate_1 in Figure 9).

It can be seen that the vibration amplitude of the contact point located on the exciting bimorph plates is 1.33 times larger than the other two contact points when the actuator does not have an external load and operates at the resonant frequency (Figure 9a). The displacement amplitude of the active contact point is 534.5 µm, while the other two rods generate displacement amplitudes of 401.6 and 396.9 µm. By analyzing vibrations amplitudes of the rods when an external load of 546.03 m N is applied, it can be seen that the difference between amplitudes increases up to 9.61 times (Figure 9b). The displacement amplitude of the contact point located on the active plate is 108.6 µm, while the other two rods generate displacement amplitudes of 9.5 and 11.3 µm. Simulation results show that the vibration amplitudes of the rods located on the passive bimorph plates are reduced significantly when the external load is applied. Based on simulation results, it can be concluded that vibrations of the passive rods push the rotor or stage in the opposite direction compared to the active rod, but the force they generate is significantly smaller than that of the active rod.

**Figure 10:**
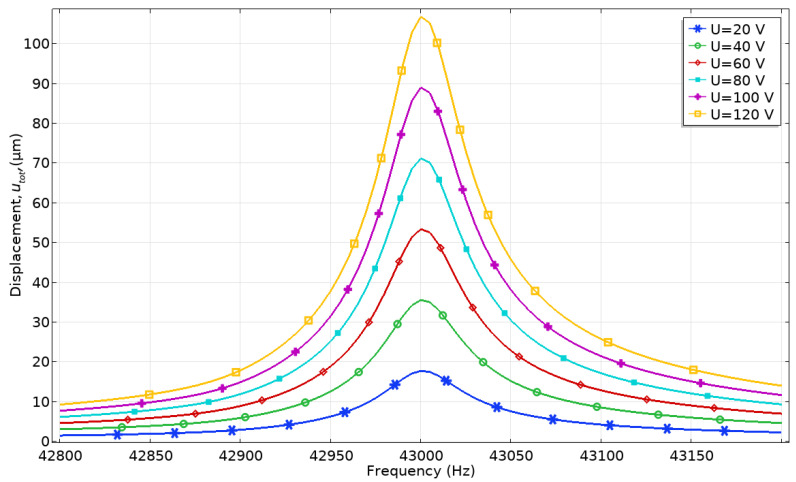
Displacement amplitude-frequency characteristics at different excitation voltages.

**Figure 11:**
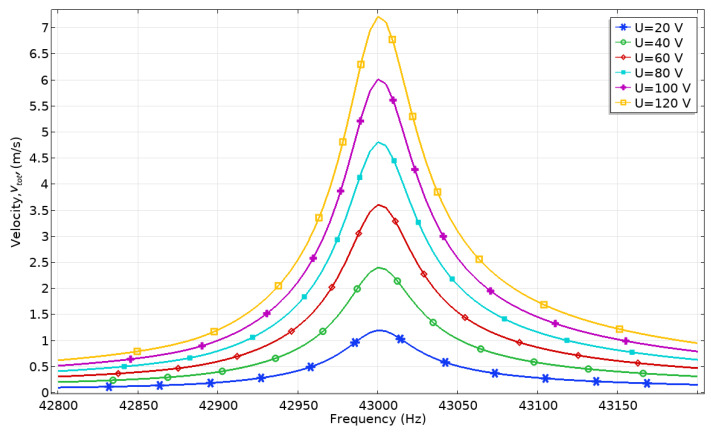
Velocity-frequency characteristics at different excitation voltages.

The dependence of the contact point displacement and velocity amplitudes from excitation voltage was analyzed as well. Excitation voltage from 20 to 120 V_p-p_ with the step of 20 V_p-p_ was applied while the frequency range was set from 42.8 to 43.4 kHz with a step resolution of 2.5 Hz. Voltage was applied only to the electrodes of one bimorph plate. The numerical model with an external load of 546.03 mN was used. The results of calculations are given in Figure 10 and Figure 11.

The displacement amplitude-frequency characteristic shows that displacement amplitude peaks are obtained at the frequency of 43.05 kHz. The lowest displacement amplitude of 17.8 µm is obtained when the voltage of 20 V_p-p_ is applied, while the highest amplitude of 108.61 µm is obtained at 120 V_p-p_. The ratio between contact point displacement amplitude and the applied voltage is 0.89 and 0.905 µm/V_p-p_, respectively.

The maximum velocity of contact point motion is obtained at the same frequency of 43.05 kHz (Figure 11). The lowest velocity amplitude is obtained at 20 V_p-p_ and reached 1.21 m/s or 0.06 m/s/V_p-p_, while the highest velocity amplitude is obtained at 120 V_p-p_, and it reached 7.22 m/s or 0.0601 m/s/V_p-p_. Based on the results, it can be stated that the actuator has a nearly linear dependence of displacement and velocity amplitudes from excitation voltage; therefore, the dynamic characteristics of the actuator can be easily controlled.

**Figure 12:**
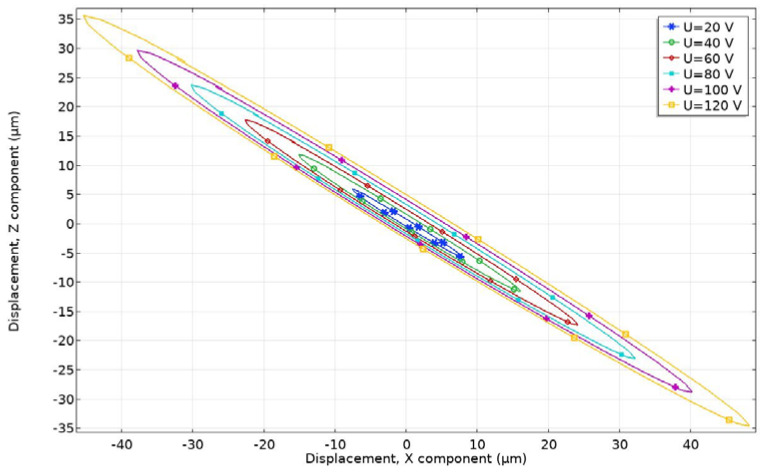
Motion trajectories of contact point at different excitation voltage amplitudes.

A numerical study of contact point motion trajectories at different excitation voltages was performed in order to confirm the operating principle of the actuator. The size and shape of the elliptical trajectory, as well as length of ellipsis axes, have an important effect on the contact conditions and affect the power distribution mechanism and friction time-varying process. Excitation voltage was applied to the single bimorph plate in a range from 20 to 120 V_p-p_, with the step of 20 V_p-p._ The time-domain study was performed at the frequency of 43.05 kHz. The analyzed time interval was equal to one cycle (T) of actuator vibration, while the time step was T/100. The period of actuator vibration is equal to 23.23 µs, and the time step is 232.3 ns. Analyzing the results, it can be seen that contact point motion has an elliptical trajectory. When a harmonic signal with a frequency between the B_20_ and B_03_ modes is applied, the bending and radial modes respond asynchronously with a phase difference due to the structural damping of the stator. The length of the major and minor axis increases when excitation voltage is increased (Figure 12). This means that contact motion velocity increases as well. The shortest length of the major axis of 19.43 µm was obtained at a voltage of 20 V_p-p_, while the largest length of the major axis is 115.35 µm, and it was obtained when the voltage of 120 V_p-p_ was applied. The inclination angle of the major ellipsis axis is 143.4 degrees in all cases. Dimensions of the elliptical motion can be changed by changing excitation frequency when a single electric signal is used for actuator excitation. The length of the major and minor axes affects the geometrical form of the contact and the start-up and stable running of the motor, while the inclination angle influences contact width and affects rotor sliding. 

**Figure 13:**
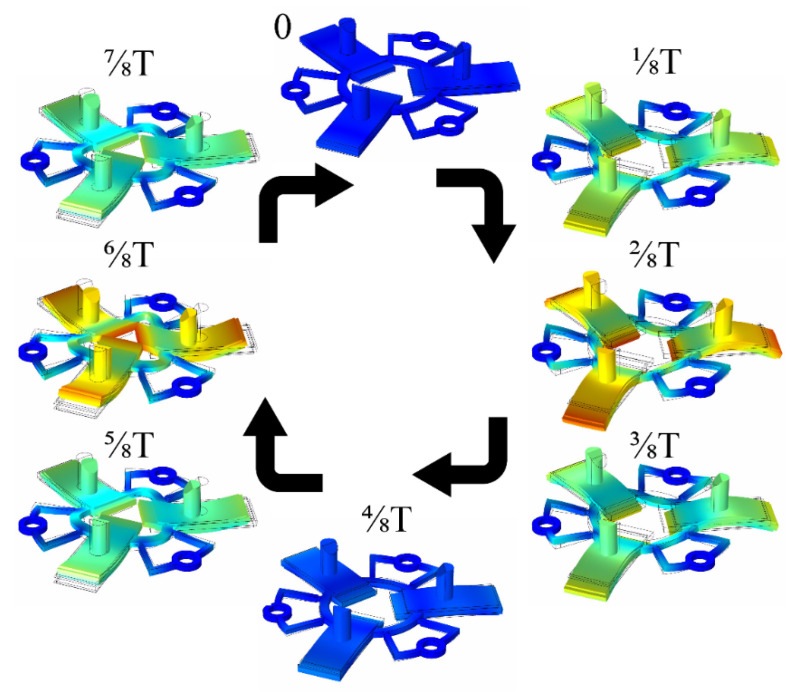
The operation sequence of the actuator during one period of vibrations.

Vibrations of the actuator were simulated at the excitation frequency of 43.05 kHz during one time period (T) when all bimorph plates were excited by the voltage of 120 V_p-p_. This simulation was performed to show the vibration shapes of the actuator at every 1/8 T and validate the operating principle of the actuator (Figure 13). Results show that during the first quarter of the oscillation period, the bimorph plates bend upwards, and at the same time, the ring expands in the radial direction. The plate and ring shape returned to their original position during the second quarter of the period. In the third quarter of the period, the plates bend downwards, and the ring contracts to the center. Later, the shape of the actuator returns to its original position. It can be concluded that the vibrations of the actuator combine the bending mode of the plate and radial mode of the ring, while the contacting point of the rod moves in an elliptical trajectory.

Results of the numerical investigation confirmed that the proposed design of the actuator can be used to generate the rotary motion of the spherical rotor as well as the liner motion of the flat stage. Moreover, optimized geometrical parameters allowed minimizing the difference between resonant frequencies of B_20_ and B_03_ vibration modes. The obtained results show that the actuator has a nearly linear dependence of the displacement and velocity from the applied voltage.

## 4. Experimental Investigation of the Motor

**Figure 14:**
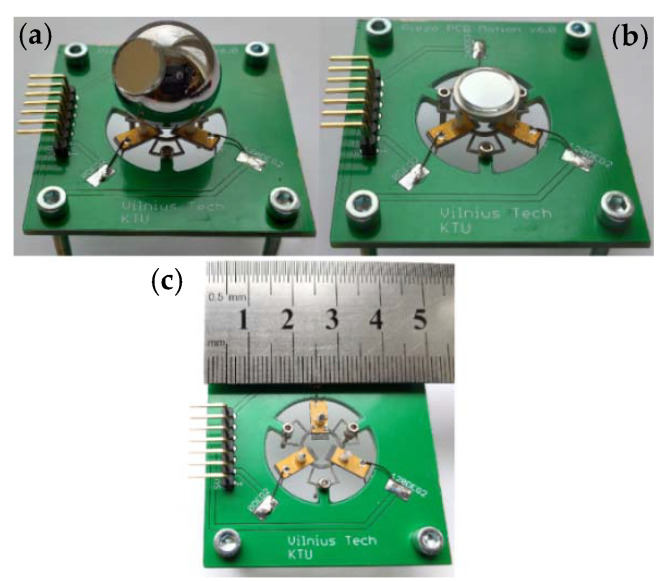
A prototype of the actuator; (**a**)—actuator composed with the spherical rotor; (**b**)—actuator composed with planar stage; and (**c**)—actuator without active part.

An experimental investigation of the actuator was performed to validate the operating principle of the actuator and to measure electrical and mechanical output characteristics. A prototype of the actuator was made with strict respect to geometrical parameters as well as boundary conditions used during the numerical study. The top view of the prototype actuator integrated into the printed circuit board with the rotor and flat stage is shown in Figure 14.

**Figure 15:**
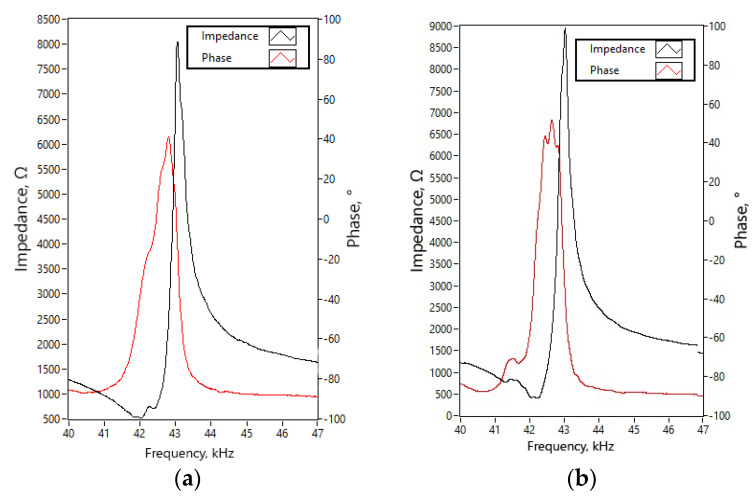

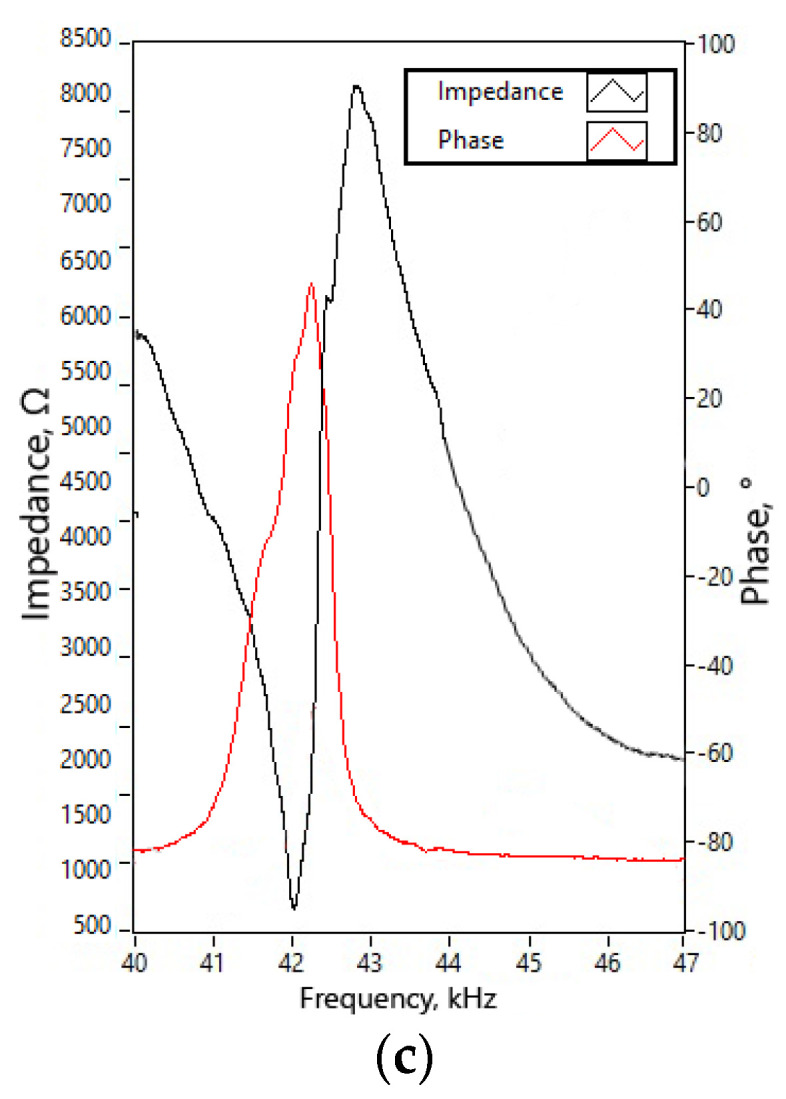
The measured impedance and phase characteristics in the frequency domain; (**a**)—bimorph plate No.1; (**b**)—bimorph plate No.2; and (**c**)—bimorph plate No.3.

Impedance–frequency and phase-frequency characteristics of the actuator were measured using impedance analyzer SinPhase 16777k (SinePhase Instruments GmbH, Austria). Measurement of each bimorph plate was measured separately. The actuator was clamped into PCB, and both piezoceramic plates of the bimorph plate were connected in parallel, while the other two bimorph plates were set to open-circuit conditions. Measurements were made without the rotor. The mechanical boundary conditions fully corresponded with the numerical model. The results of measurements are given in Figure 15. The summary of the measured resonant frequency, impedance value, and a mechanical quality factor and the effective coupling coefficient, is given in Table 5. 

By analyzing the measured resonant frequencies of the three bimorph plates, it can be noted that small differences of up to 3.1% can be observed. A comparison of calculated and measured resonant frequencies revealed that the highest difference between values is 1.315 kHz or 3.15%.

The lowest measured impedance of 518.3 Ω was obtained. It is a 61.3% lower value compared to the results of the numerical study. The difference between numerically obtained and experimentally measured values of mechanical quality factor and effective coupling coefficient is up to 38.1% and 27.6%, respectively. The mismatch of the values is caused by the glue layer that was neglected during numerical investigations, minor differences in material characteristics, and differences of actuator clamping conditions during experimental and numerical investigations. On the other hand, a comparison between calculated and measured values shows that results have an acceptable agreement, and the prototype of the actuator can be further investigated.

**Figure 16:**
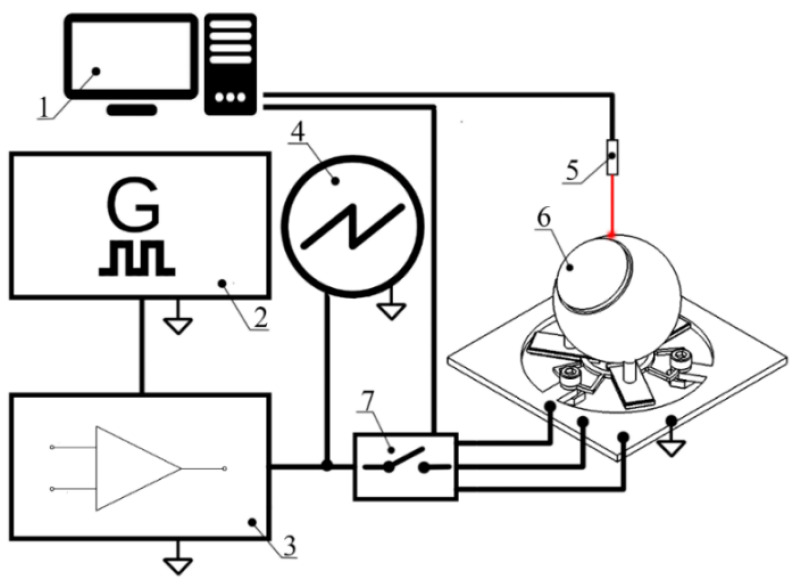
Scheme of the experimental setup: 1—a computer with data acquisition and switch control software; 2—function generator; 3—power amplifier; 4—oscilloscope; 5—noncontact tachometer; 6—a prototype of the actuator; and 7—digitally controlled switch box.

**Figure 17:**
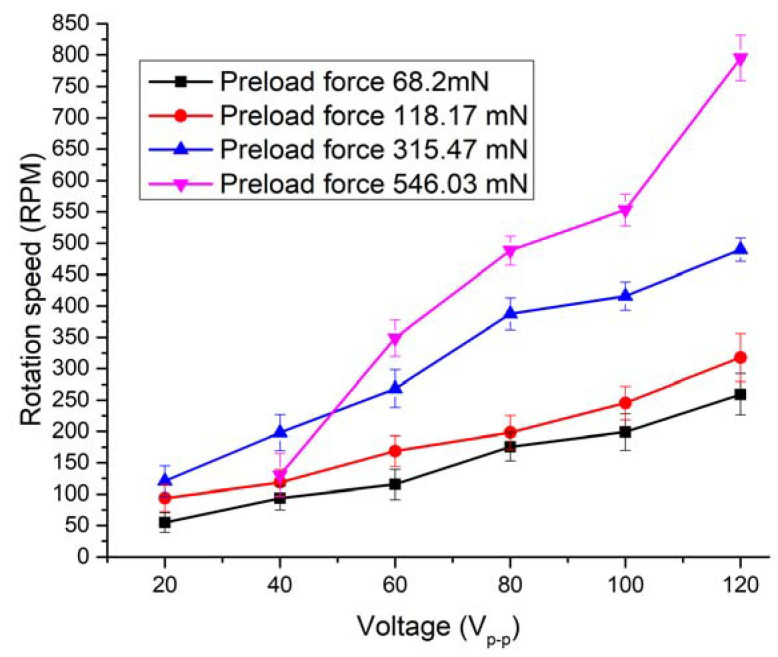
The rotation speed of spherical rotor at different excitation voltages and preload forces when bimorph plate No. 2 is excited.

**Figure 18:**
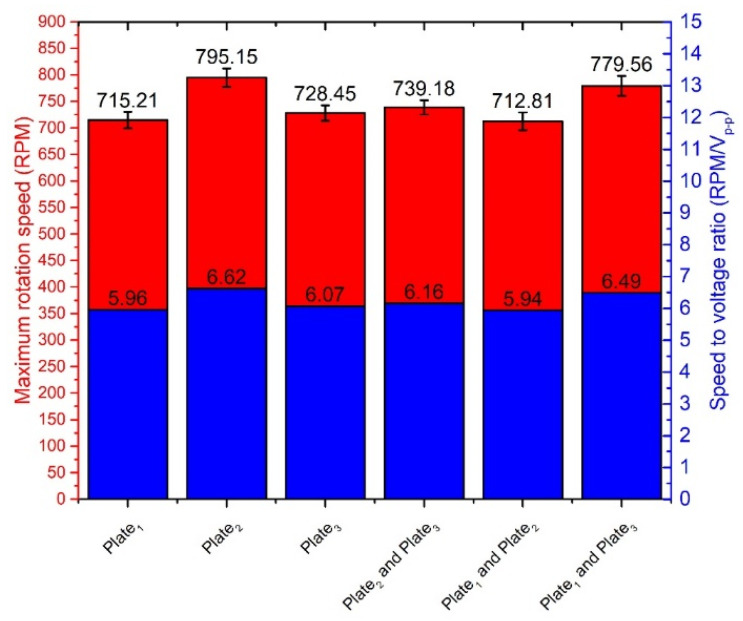
Comparison of the rotation speeds and speed to voltage ratio.

Measurements of the rotation speed and linear velocity were performed to investigate the dynamic characteristics of the actuator at a different excitation voltage. The experimental setup was built for this purpose (Figure 16). The experimental setup consisted of a computer, a function generator WW5064 (Tabor Electronics, Israel), a power amplifier PX—200 (Piezo Drive, Australia), oscilloscope DL2000 (Yokogawa, Japan), a noncontact tachometer CA 1727 (Chauvin Arnoux, France), and a custom-made switch box. The rotation speed and linear velocity of the active part were measured by varying the excitation voltage from 20 to 120 V_p-p_. In addition, four different preload force values were applied. The detailed experimental study was performed with the bimorph plate No. 2 because it has the lowest impedance value. The remaining two bimorph plates were investigated only when an excitation voltage of 120 V_p-p_ was applied. The results of plate No. 2 measurement are given in Figure 17 and Figure 18. In addition, Videos S1–S3, were included as a supplementary material in order to show actuator operation while different plates are excited. Moreover, Video S4 shows actuator operation in step mode.

By analyzing the results of the measured rotation speed, it can be seen that the lowest value of 54.6 RPM was obtained at the preload force of 68.2 m N and voltage of 20 V_p-p_ (Figure 17). On the other hand, the rotation speed of 259.1 RPM was reached at the same preload when the voltage was increased up to 120 V_p-p_. The average speed–voltage ratio is 2.66 RPM/V_p-p_.

The highest rotation speed of 795.15 RPM was obtained when plate No.2 was excited, and preload force was 546.03 m N, and voltage of 120 V_p-p_ was applied. The ratio between speed and voltage is 6.62 RPM/V_p-p_ in this case. However, the starting voltage is 40 V_p-p_ at this preload value, and rotation speed is 130.3 RPM or 3.26 RPM/V_p-p_, respectively. The starting voltage of the actuator was 20 V_p-p_ at other preload force values. Results showed that that rotation speed has an almost linear dependence from the voltage. The comparison of the measured rotation speed and speed to voltage ratio is shown in Figure 18, when different single bimorph plates and pairs of the bimorph plates described as cases 4, 5, and 6 in Table 2 are excited. Measurements were made when excitation voltage of 120 V_p-p_ and preload of 546.03 mN was applied. The difference between obtained results does not exceed 82.34 RPM or 10.35%. The differences are mainly caused by minor different resonant frequencies as well as minor differences in contact conditions between the spherical rotor and cylinder-shaped beams used to transfer the vibration from plates to the active part of the actuator.

**Figure 19:**
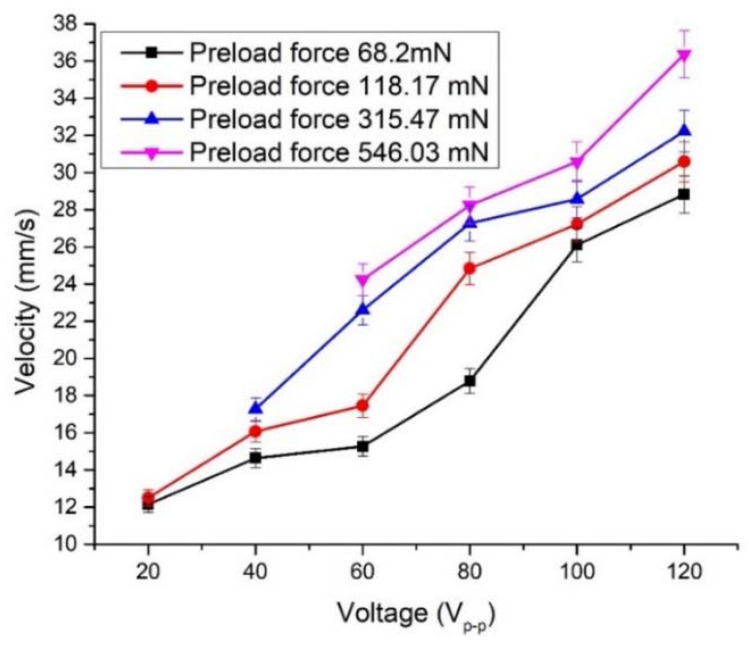
The linear velocity of the flat stage at different preload forces and voltages when bimorph plate No.2 is excited.

The linear velocity measurement of the planar stage was performed as well. Detailed measurements were made with plate No. 2, as was done in the previous case. In addition, as supplementary information, Video S5 was included in order illustrate actuators operation while different plates were excited. Therefore, the lowest linear velocity was obtained when preload force was 68.2 mN and a voltage of 20 V_p-p_ was used (Figure 19). The linear velocity was equal to 12.14 mm/s or 0.6 mm/s/V_p-p_. When excitation voltage was increased up to 120 V_p-p_, the linear velocity of 28.82 mm/s or 1.44 mm/s/V_p-p_ was achieved. It must be pointed out that starting voltage increases when preload is increased, i.e., the actuator begins to generate linear motion at the input voltage of 40 V_p-p_ when preload force of 315.45 mN is applied. Accordingly, starting voltage is 60 V_p-p_ when preload is 546.03 mN. Comparing measured characteristics of rotary and linear motion, it can be observed that the actuator is more sensitive to the preload force when it operates in linear motion mode. This can be explained because of differences in contact zones and preload force interaction type with the actuator.

The highest linear velocity values were obtained when the preload force value was 546.03 mN. The actuator achieved a velocity of 24.25 mm/s at the voltage of 60 V_p-p_ and 36.45 mm/s at the voltage of 120 V_p-p_. The ratio between velocity and applied voltage was 0.404 and 0.305 mm/s/V_p-p_, respectively.

**Figure 20:**
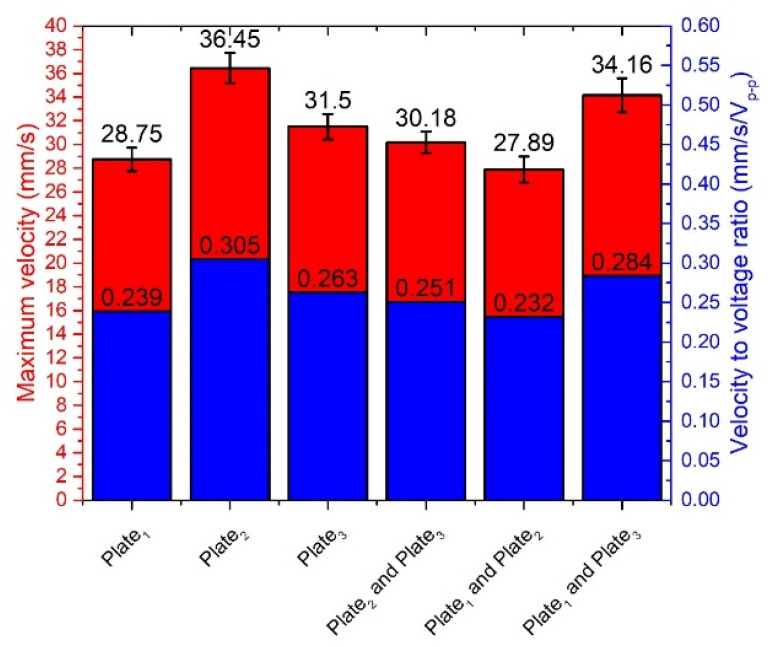
Comparison of linear velocity and velocity-to-voltage ratio of the flat stage.

The comparison of the measured linear velocity and velocity-to-voltage ratio is shown in Figure 20, when different single bimorph plates and pairs of the bimorph plates described as cases 4, 5, and 6 in Table 2 are excited. Measurements were made when excitation voltage of 120 V_p-p_ and preload of 546.03 m N was applied. The difference between the obtained results does not exceed 8.56 mm/s or 23.48%.

**Figure 21:**
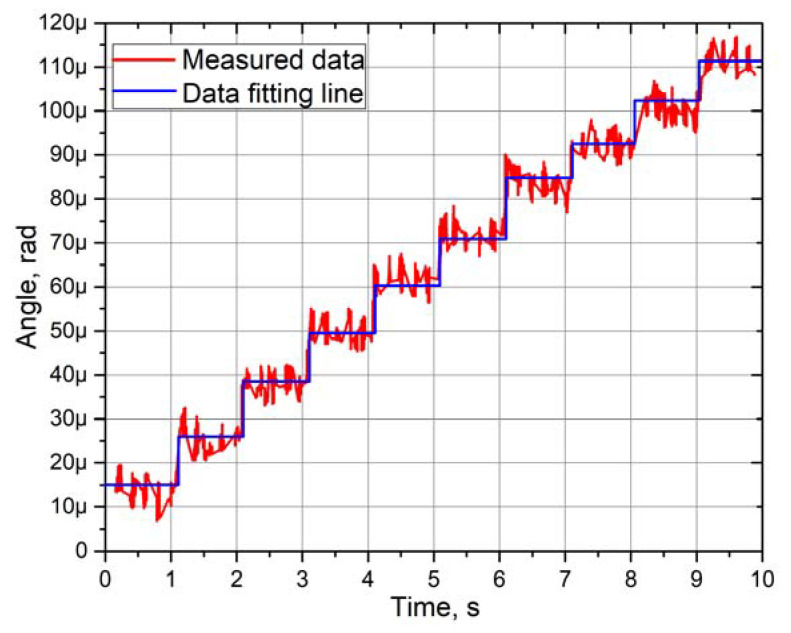
Angular motion resolution characteristics of the actuator.

**Figure 22:**
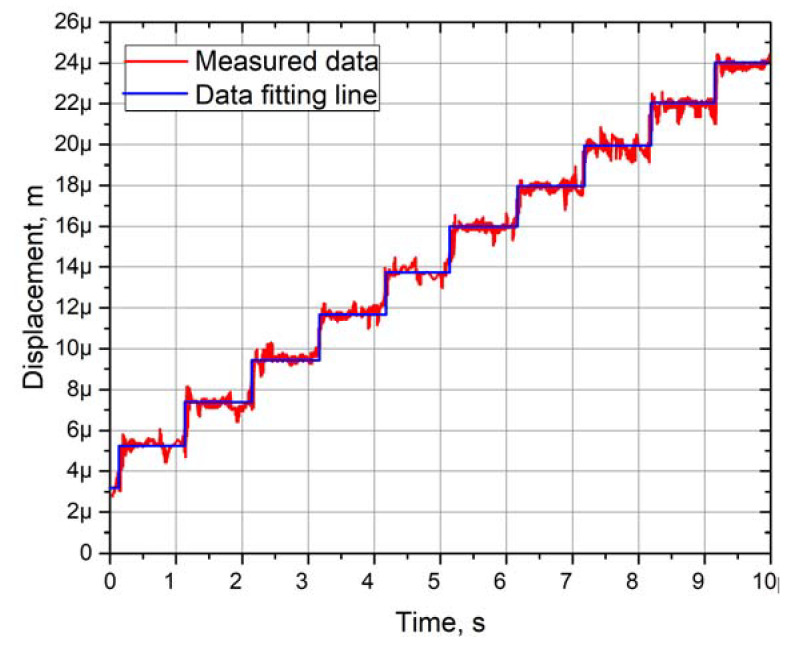
Linear motion resolution characteristics of the actuator.

**Figure 23:**
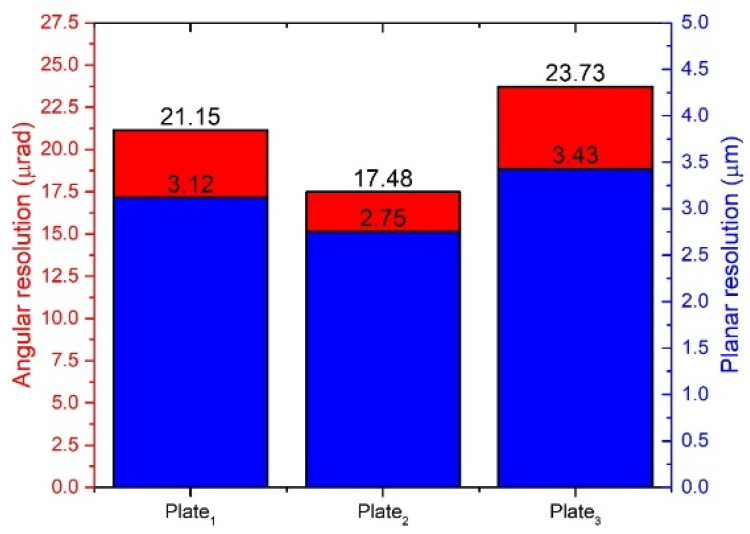
Summary of angular and linear resolution values.

Experimental measurements of the angular and linear resolution of the actuator were performed using a 3D Polytec vibrometer (Polytec, Germany). Burst-type harmonic excitation signal consisting of 20 cycles was used to drive one bimorph plate, i.e., SW_1_ was turned on, while SW_2_ and SW_3_ were turned off (Figure 3). Preload force was set to 118.17 mN. This arrangement of excitation signal ensures stepping type motion of active part of the actuator. Moreover, the maximum burst-type signal amplitude was limited to 120 V_p-p_. The results of measurement of the bimorph plate No.2 are shown in Figure 21 and Figure 22, while the comparison of the other two plates’ measurement is shown in Figure 23.

The angular resolution reached 17.48 µrad as a maximum value, while the minimum value is 12.78 µrad (Figure 21). The deviation between two adjacent steps is approximately from 1 to 4 µrad. However, these values are based on fitting line results and should be considered as average angular resolution characteristics. In addition, the measured data show that the rotor has a minor angular motion deviation range from 5 to 7 µrad. These deviations can be reduced by reducing the maximum amplitude of harmonic burst-type excitation signal. However, it affects the step resolution and causes the need to reduce preload force that reduces resolution as well.

Linear motion resolution of the flat stage was investigated at the same conditions as angular (Figure 22). The spherical rotor was changed to a flat stage with preload force of 118.18 mN. The analysis of the results showed that the minimum resolution of linear motion is 1.89 µm, while the maximum value is 2.75 µm. The deviation between two adjacent steps is approximately from 1 to 1.5 µm. However, these values are based on fitting line results and are averaged as in the case with angular resolutions. Analysis of measured values revealed that the flat stage generates parasitic motion between steps, and it deviates in the range from 0.5 to 2 µm. Compares to the angular resolution measurement, these parasitic motions are notably lower.

Analyzing the results shown in Figure 22 it can be noted that the difference between the angular resolution of the bimorph plates does not exceed 6.25 µrad or 26.34% and 0.68 µm or 19.82% for linear resolution. The resolution differences are caused by manufacturing errors, differences in material properties, and adhesive layer.

Based on the experimental study results, it can be concluded that the actuator can generate high-speed rotational and linear motion. Almost linear output speed depends on excitation voltage, and starting voltage value depends on the preload force. On the other hand, results showed the actuator could operate at low voltage as well. Moreover, the actuator can achieve microscale angular and linear resolution by applying burst-type electric signal. 

Finally, a comparison of the proposed actuator to state-of-the-art piezoelectric actuators was performed and is shown in Table 6. The comparison includes rotary–linear type piezoelectric actuators. The operating principle of the actuators was not taken into account. An analysis of the values given in Table 6 shows that the proposed actuator has the highest maximum rotation speed and smallest angular resolution. In addition, it achieves a high linear velocity that is smaller only for the actuator reported by Mashimo and Toyama [23]. The linear resolution of the proposed actuator is larger compared to the other actuators; nevertheless, the resolution range is similar.

## 5. Conclusions 

Notably, 2-DOF rotary–linear motion actuator was developed and investigated. The design of the actuator is scalable and suitable for mount on the PCB as well as use for applications where mounting space is limited. The numerical and experimental investigation was performed, and the following conclusions were formulated.

1. Superposition of bimorph plate bending mode B_20_ and radial mode B_03_ of the ring can be achieved by changing the length of the bimorph plate and the diameter of the ring.

2. The optimal location of the rod on the top surface of the bimorph plate allows one to increase vibration amplitudes significantly. The maximum displacement amplitude value of 108.61 µm and velocity value of 7.22 m/s were obtained at the frequency of 43.05 kHz, while excitation voltage was set to 120 V_p-p_.

3. Experimental investigations confirmed the operation principle of the actuator and validated that rotational and linear motion of the rotor and flat stage can be induced by applying the same vibration mode of the actuator.

4. The maximum measured rotation speed of the rotor reached 795.15 RPM, while the maximum linear velocity of the flat stage was 36.45 mm/s when excitation voltage of 120 V_p-p_ was applied. The differences of the rotational and linear velocity when different bimorph plates are excited do not exceed 10.1% and 21.12%, respectively.

5. Resolution of angular motion of 17.48 µrad and linear motion resolution of 2.75 µm was obtained. The resolution differences of angular and linear motion of different bimorph plates do not exceed 26.34% and 19.82%, respectively.

6. Excitation of the actuator can be performed using a single harmonic signal or a digitally controlled switch box. More sophisticated excitation schemes consisting of two or three electric signals may also be used.

7. A study of the proposed actuator revealed several drawbacks. Impedance measurements showed that bimorph plates have different resonant frequencies because of manufacturing errors. Therefore, control of the actuator becomes more complex. The numerical study showed that all three rods vibrate even if a single bimorph plate is excited. It means that two rods located on non-excited plates drive the spherical rotor or stage in the opposite direction compared to the driving direction of the active rod, although the displacements of these two rods are minor. In order to solve this problem, the actuator should be revised by including passive or active vibration damping of non-excited bimorph plates.

## Figures and Tables

**Table 1:** Geometrical parameters of stator.

Parameter	Value	Description
L_plate_	9.33 mm	Length of stainless-steel plate
L_pzt_	8 mm	Length of piezo ceramic plate
L_Clamp_	3.9 mm	Length of U-shaped support
L_Beam_	3 mm	Length of cylinder shaped beam
W_Plate_	4 mm	Width of stainless-steel plate
W_pzt_	3.6 mm	Width of piezo ceramic plate
W_Ring_	0.8 mm	Width of ring
W_Clamp_	0.5 mm	Width of U-shaped support
R_Hinge_	0.18 mm	Radius of flexible hinge
R_Beam_	0.75 mm	Radius of cylinder shaped beam
D_Clamp_	1.4 mm	Diameter of clamping spot
D_Total_	22.5 mm	Diameter of whole stator
D_Ring_	8.46 mm	Centre diameter of ring
t_total_	0.9 mm	Total thickness of bimorph plate
t_pzt_	0.2 mm	Thickness of piezo ceramic plate
t_plate_	0.5 mm	Thickness of stainless-steel plate
α	120°	Distribution angle of bimorph plates
β	45°	Inclination angle of U-shaped support
γ	225°	Ange of contact zone inclination
S	391.5 mm^2^	Surface area of the stator

**Table 2:** Switch positions for motion direction control.

Case No.	Direction	SW_1_	SW_2_	SW_3_
1	0°	1	0	0
2	60°	0	0	1
3	120°	0	1	0
4	180°	0	1	1
5	240°	1	1	0
6	300°	1	0	1

**Table 3:** Materials properties.

Material Properties	Stainless-Steel ISO A2	PI Ceramics PIC181	Aluminum Oxide Ceramic
Density, [kg/m^3^]	8000	7800	3980
Young’s modulus, [N/m^2^]	10 × 10^9^	7.6 × 10^10^	41.9 × 10^10^
Poisson‘s coefficient	0.3	-	0.33
Isotropic structural loss factor	0.02	-	0.2 × 10^−3^
Relative permittivity	-	ε_11_^T^/ ε_0_ = 1200 ε_33_^T^/ε_0_ = 1500	-
Elastic compliance coefficient [10^−12^ m^2^/N]	-	S_11_^E^ = 15.00 S_33_^E^ = 19.00	-
Elastic stiffness coefficient c_33_^D^, [N/m^2^]	-	1.6 × 10^10^	-
Piezoelectric constant d_33_ [10^−12^ m/V]	-	225	-
Piezoelectric constant d_31_ [10^−12^ m/V]	-	−97	-
Piezoelectric constant d_15_ [10^−12^ m/V]	-	330	-

**Table 4:** Optimal values of variables and values of the objective functions.

*L_Plate_*, mm	$D_{Ring}^{},\mathrm{mm}$	*L_pos_*, mm	Δ*f*, Hz	*u_tot_*, µm
9.33	8.46	4.25	590	113.5

**Table 5:** Results of impedance and frequency measurement.

Bimorph Plate	Frequency, kHz	Impedance, Ω	*Q_m_*	*k_eff_*
Plate No.1	42.03	548.1	1798.1	0.0348
Plate No.2	42.35	518.3	1754.3	0.034
Plate No.3	42.17	625.8	1801.3	0.0351

**Table 6:** Comparison of the piezoelectric rotary—linear actuators.

Authors	Excitation Voltage (V_p-p_)/ Frequency (kHz)	Maximum Rotation Speed (RPM)	Maximum Linear Speed (mm/s)	Resolution of Linear Motion (µm)	Resolution of Angular Motion (µrad)
Mashimo and Toyama [23]	42/305	228.96	80	N/A	N/A
Rakotondrabe and et al. [25]	150/10	3.32	1.8	0.2	43.45
Han and et al. [24]	720/2–11	9.44	2.4	1.03	1000
Sun and et al. [6]	140/0.15	3.26	1.45	9.3	228.5
Proposed actuator	120/42.35	795.15	36.45	3.43	23.73

## Supplementary Materials

The following are available online at https://www.mdpi.com/article/10.3390/mi12060728/s1; Video S1: represents the rotation of the spherical rotor while Plate_1 is excited; Video S2 represents the rotation of the spherical rotor while Plate_2 is excited; Video S3 represents the rotation of the spherical rotor while Plate_3 is excited; Video S4 represents the rotation of the spherical rotor in step mode; and Video S5 represents actuator operation in planar motion mode.
